# Supplementary material for: Bridging mathematical modeling and AI for 3D coordinate recognition of moving objects without external reference and attitude measurement
Source: Commun Eng. 2026 Mar 20;5:89. doi: 10.1038/s44172-026-00648-x (PMC13168241; doi:10.1038/s44172-026-00648-x)
Supplement: Supplementary file 2 — Supplementary Information.pdf [file 44172_2026_648_MOESM2_ESM.pdf]

Bridging Mathematical Modeling and AI for 3D Coordinate Recognition of Moving Objects without External Reference and Attitude Measurement

Junfan Yi[1,2,3†], Ke-ke Shang[2*†], Michael Small[1,4]

[1]Complex Systems Group, Department of Mathematics and Statistics, The University of Western Australia, Crawley, Perth, 6009, WA, Australia.

[2*]Computational Communication Collaboratory, Nanjing University, Qixia, Nanjing, 210023, Jiangsu, China.

[3]The School of Geography and Ocean Science, Nanjing University, Qixia, Nanjing, 210023, Jiangsu, China.

[4]Mineral Resources, CSIRO, Kensington, Perth, 6151, WA, Australia.

*Corresponding author(s). E-mail(s): kekeshang@nju.edu.cn, keke.shang.1989@gmail.com;
Contributing authors: junfan.yi@research.uwa.edu.au; michael.small@uwa.edu.au;
†These authors contributed equally.

Supplementary Information

S1 Supplementary Figures

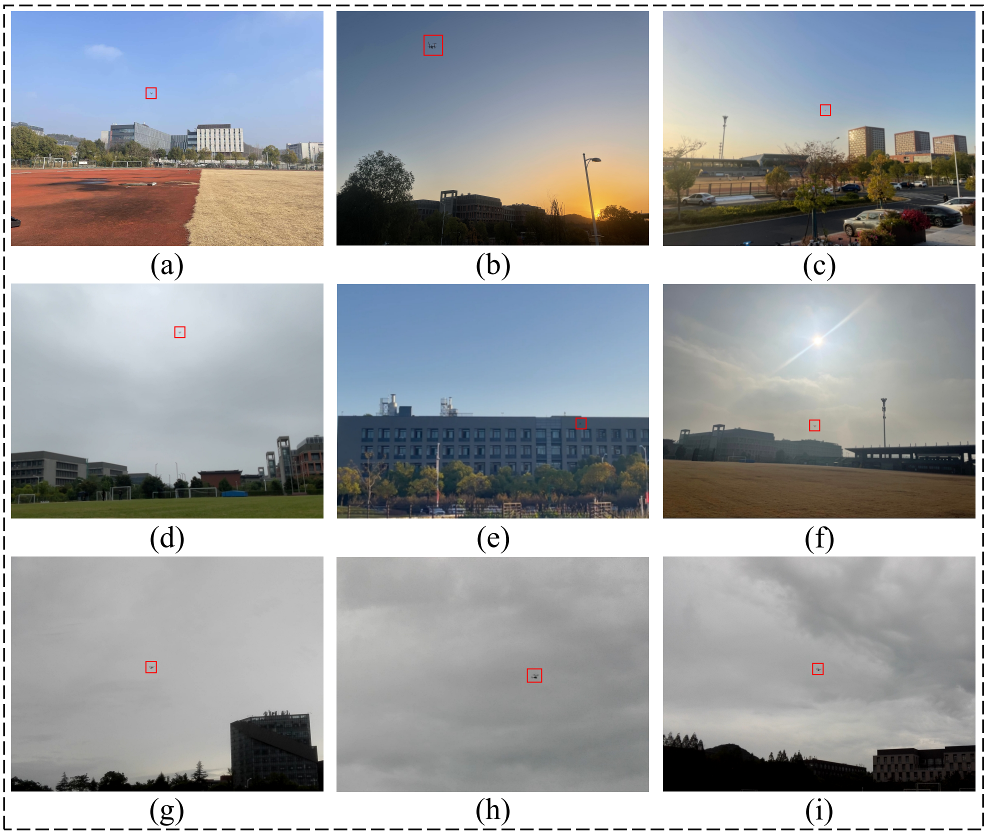

Supplementary Figure 1 Images of a flying UAV captured by ground cameras under various background conditions. With UAV detections highlighted by red rectangles. Backgrounds include (a) clear, cloudless sky, (b) dusky, dark sky, (c) afternoon sky, (d) cloudy sky, (e) buildings in the background, (f) backlighting, and (g)–(i) rainy conditions with reduced visibility. Photos taken by the author Junfan Yi.

S2 Supplementary Discussion

Standard multi-view geometry approaches, specifically Structure from Motion (SfM) and Global Bundle Adjustment (BA), are widely used in 3D reconstruction. However, our experimental setup presents a degenerate case for these classical methods: three stationary ground-based cameras tracking a dynamic UAV against a featureless sky background. As summarized in Supplementary Table 1, both conventional approaches

fail under these conditions, while our hybrid framework succeeds. Additionally, we provide a detailed analysis of AI detector selection to justify our choice of YOLOv12 as the primary detector.

Supplementary Table 1 Comparison of Multi-view Geometry Approaches. Comparison of conventional methods with our proposed hybrid framework under real-time and featureless constraints.

Aspect	Feature-based SfM	Global BA	Ours
**Input Data**	Keypoints	Initial Pose Guess	2D Detection
**Initialization**	Fail	Fail	SVD (closed-form)
**Per-frame Complexity**	$O(N^2)$	$O(N^3)$	$O(1)$
**Featureless Scene**	Fail	Fail	Works
**Real-time**	Fail	Fail	Works

S2.1 Baseline A: Failure of Conventional Feature-based SfM

We attempted to employ standard SfM pipelines to determine if they could determine the UAV 3D coordinate without semantic priors. However, standard SfM is inapplicable to our problem due to a fundamental inversion of the observation model: SfM assumes a moving camera observing a static scene, whereas our scenario involves stationary cameras observing a dynamic object. Three specific issues arise:

Feature Starvation on Textureless Targets. SfM relies on local descriptors (SIFT/ORB) to find correspondences. In our setting, texture extraction from the target is difficult due to its small size, and the background is dominated by sky. Clouds are not only dynamic but also deformable, violating the rigidity assumption. In our experiments, the number of detectable feature points on the UAV surface was frequently zero or insufficient for epipolar geometry estimation, preventing solver initialization (Supplementary Figure 2).

Rejection of the Dynamic Target. SfM assumes that the scene is static. In our scenario, the UAV is the only moving object. During geometric verification, algorithms classify moving target features as outliers to preserve background consistency. Consequently, standard pipelines discard the UAV and reconstruct only the static background.

The Stationary Camera Configuration. Our cameras are fixed on the ground. Classical SfM relies on camera motion to recover 3D structure from video sequences. Since our cameras do not move, no geometric parallax is generated over time. This results in a degenerate configuration where the solver cannot triangulate the moving target, because the system lacks the camera-scene relative motion required for reconstruction.

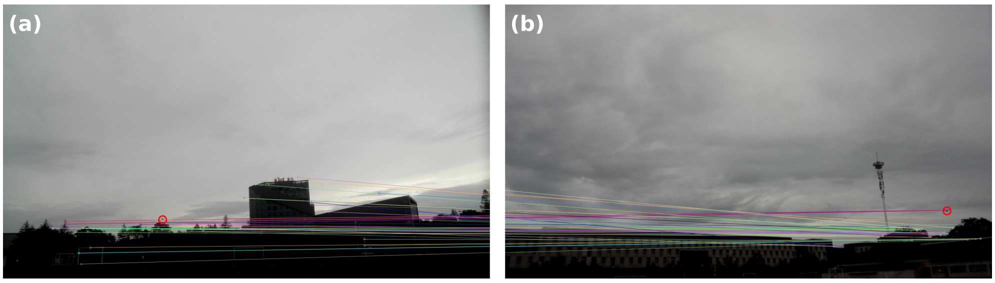

Supplementary Figure 2 Visualization of SfM Feature Matching Failure. (a) and (b) represent two frames from the stationary camera sequence. Red circles indicate the ground-truth position of the target UAV. The colored lines represent computed feature matches. Note the phenomenon of "Feature Starvation". The geometric feature matcher fails to identify any valid key points within the UAV region (marked by red circles). Matches are exclusively concentrated on the static background (trees and buildings), and many are mismatched (crossing lines) due to the non-rigid nature of the vegetation. The sky region remains completely devoid of features. Consequently, the SfM solver cannot recover the UAV's motion. Photos taken by the author Junfan Yi.

S2.2 Baseline B: Infeasibility of Pure Global Bundle Adjustment

Global Bundle Adjustment (BA) is widely regarded as the "Gold Standard" for high-precision estimation. Theoretically, it provides the Maximum Likelihood Estimate by minimizing the reprojection error over all historical frames. In our architecture, we incorporate Global BA as a background module to use this optimality.

However, executing Global BA synchronously (frame-by-frame in the control loop) is infeasible due to two constraints:

The Initialization Barrier. BA is a non-linear optimization process that requires a high-quality initial guess. The cost landscape is non-convex. Without external attitude sensors (e.g., IMU), a standalone BA solver initialized with random values cannot converge. This is known as the cold start problem.

The Latency Barrier. Even with perfect initialization, synchronous Global BA violates the hard real-time requirement. As the mission duration increases, the accumulated observations N grow, and the computational complexity (Hessian inversion) scales cubically, $O(N^3)$. As shown in Supplementary Table 1, the optimization time for Global BA exceeds the real-time threshold, blocking the high-frequency control loop.

Strategic Solution: Dual-Phase Strategy with Background Refinement. To combine the high precision of BA with strict real-time constraints, we designed a dual-phase strategy that decouples refinement from real-time tracking. During Phase II (Online Tracking), the system uses SVD-based triangulation, which operates in constant $O(1)$ time per frame. This guarantees low latency and provides initial pose estimates that bypass the initialization barrier. The Background Refinement Module executes sliding-window Bundle Adjustment at fixed intervals without blocking real-time processing. By using system idle time to refine the accumulated observations, the system achieves increasingly accurate pose estimates without compromising real-time performance.

S2.3 AI Detector Selection and Sensitivity Analysis

Rationale for detector selection. The proposed architecture operates in a hard real-time environment designed to track uncooperative UAVs without external attitude measurements. Given the camera input frequency of 30 FPS, the inter-frame interval is approximately 33 ms. To ensure valid online tracking without frame buffering, the total end-to-end latency must remain below this 33 ms budget. This temporal constraint serves as the primary exclusion criterion for detector selection. Two-stage detectors, such as Faster R-CNN and Mask R-CNN, were excluded because they prioritize detection accuracy over inference speed, resulting in latencies that violate the real-time constraint. Similarly, detection transformers (e.g., DETR) were excluded; variants capable of detecting small aerial targets (such as DETR-DC5) require high-resolution feature maps, introducing computational overhead incompatible with online processing. Consequently, we focus on single-stage detectors, selecting the YOLO family to validate the proposed hybrid framework.

Experimental setup and model architecture. We used the official Ultralytics implementation (https://github.com/ultralytics/ultralytics) to ensure reproducibility. To maintain a consistent baseline within the real-time constraint, we selected the smallest variant for each version: YOLOv8n, YOLOv9t, YOLOv10n, YOLO11n, and YOLOv12n. The architectural evolution across these versions represents a shift from purely convolutional efficiency to attention-driven precision. YOLOv8 introduced an anchor-free detection head and C2f modules for improved gradient flow. YOLOv9 incorporated Programmable Gradient Information (PGI) and the Generalized Efficient Layer Aggregation Network (GELAN) to reduce information loss in deep layers. YOLOv10 focused on latency reduction through NMS-free training via consistent dual assignments. YOLO11 integrated C3k2 blocks and spatial attention mechanisms (C2PSA) for enhanced feature extraction. YOLOv12 adopts an attention-centric design with FlashAttention and R-ELAN to improve feature focus without compromising speed.

Sensitivity analysis from 2D detection to 3D reconstruction. The objective of this analysis is to determine whether the proposed framework depends on a specific model version or generalizes across detector quality levels. As shown in Supplementary Table 2, the framework exhibits stability across all tested detectors. Even with the older YOLOv8n, the system produces valid 3D coordinates, albeit with higher error. As $\text{mAP}_{50\text{-}95}$ improves from v8n to v12n, the 3D positioning error generally decreases. It proves the stability of the mathematical model while demonstrating that the framework effectively bridges the gap between AI and mathematics. YOLOv12n is selected as the primary detector because it offers the best trade-off between detection precision and inference latency.

S3 Supplementary Methods

S3.1 2D to 3D Coordinate Transformation

For two camera coordinate systems c^1 and c^2, given the rotation matrix $R_{c^2c^1}$ and the translation vector $t_{c^2c^1}$, the coordinates of point P can be transformed between

Supplementary Table 2 Impact of Detector Accuracy on 3D Positioning Error. Comparison of YOLO variants (v8n–v12n). Model parameters, $\text{mAP}_{50\text{-}95}$, and latency are official benchmarks from Ultralytics. RMSE_{3D} is the 3D positioning error from our UAV experiment

Model	Key Architecture	Params (M)	$\text{mAP}_{50\text{-}95}$ (%)	Latency (ms)	RMSE_{3D} (m)
YOLOv8n	Anchor-Free / C2f	3.2	68.4	4.2	5.66
YOLOv9t	GELAN / PGI	2.0	70.1	5.1	5.86
YOLOv10n	NMS-Free	2.3	72.5	4.8	5.60
YOLO11n	C3k2 / C2PSA	2.6	74.8	5.3	6.33
YOLOv12n	FlashAttention	2.6	76.9	5.5	5.45

c^1 and c^2 according to Equation 3 in the main text:

$$P_{c^2} = R_{c^2c^1}P_{c^1} + t_{c^2c^1}. \tag{1}$$

Given the pixel coordinates $P_{c'^1}$ and $P_{c'^2}$ of point P in the pixel coordinate systems c'^1 and c'^2, we can obtain the coordinate transformations for point P from c^1 to c'^1 and from c^2 to c'^2 based on Equation 6 in the main text:

$$Z_{c^1}P_{c'^1} = K^1P_{c^1}, \tag{2}$$

$$Z_{c^2}P_{c'^2} = K^2P_{c^2}, \tag{3}$$

where Z_{c^1} and Z_{c^2} represent the depth Z of point P in c^1 and c^2, respectively. Substituting Equation 2 and Equation 3 into Equation 1, we obtain

$$Z_{c^2}P_{c'^2} = Z_{c^1}K^2R_{c^2c^1}{K^1}^{-1}P_{c'^1} + K^2t_{c^2c^1}. \tag{4}$$

Multiplying both sides of Equation 4 on the left by the matrix $[P_{c'^2}]_\times$, we obtain:

$$Z_{c^2}[P_{c'^2}]_\times P_{c'^2} = Z_{c^1}[P_{c'^2}]_\times K^2R_{c^2c^1}{K^1}^{-1}P_{c'^1} + [P_{c'^2}]_\times K^2t_{c^2c^1}, \tag{5}$$

where $[P_{c'^2}]_\times$ is the skew-symmetric matrix of $P_{c'^2}$. For vector $P_{c'^2} = [x_{c'^2}, y_{c'^2}, z_{c'^2}]^T$, its corresponding skew-symmetric matrix $[P_{c'^2}]_\times$ is defined as follows:

$$[P_{c'^2}]_\times = \begin{bmatrix} 0 & -z_{c'^2} & y_{c'^2} \\ z_{c'^2} & 0 & -x_{c'^2} \\ -y_{c'^2} & x_{c'^2} & 0 \end{bmatrix}. \tag{6}$$

From Equation 6, we can clearly conclude that the product $[P_{c'^2}]_\times P_{c'^2}$ of a skew-symmetric matrix $[P_{c'^2}]_\times$ and its corresponding vector $P_{c'^2}$ results in a zero vector. Therefore, the left-hand side of Equation 5 equals the zero vector and we get

$$Z_{c^1} = \frac{-[P_{c'^2}]_\times K^2R_{c^2c^1}{K^1}^{-1}P_{c'^1} \cdot [P_{c'^2}]_\times K^2t_{c^2c^1}}{[P_{c'^2}]_\times K^2R_{c^2c^1}{K^1}^{-1}P_{c'^1} \cdot [P_{c'^2}]_\times K^2R_{c^2c^1}{K^1}^{-1}P_{c'^1}}. \tag{7}$$

Substituting the above solution Z_{c^1} into Equation 2, we obtain the coordinates of P in c^1:

$$P_{c^1} = \frac{-[P_{c'^2}]_\times K^2 R_{c^2c^1} K^{1^{-1}} P_{c'^1} \cdot [P_{c'^2}]_\times K^2 t_{c^2c^1}}{[P_{c'^2}]_\times K^2 R_{c^2c^1} K^{1^{-1}} P_{c'^1} \cdot [P_{c'^2}]_\times K^2 R_{c^2c^1} K^{1^{-1}} P_{c'^1}} K^{1^{-1}} P_{c'^1}. \tag{8}$$

S3.2 Detailed Derivation of 3D Coordinate Recognition

Under the condition where only the world coordinates of multiple ground-based observation cameras G^i_{w} (where $i \geq 3$, $i \in \mathbb{N}$) are available, our proposed method does not require knowledge of the camera attitude $R_{c^i\mathrm{w}}$, which is necessary for conventional methods[1] to calculate the world coordinates of O^j.

Our method decouples the coordinate recognition workflow into two phases: Batch Initialization, which recovers camera poses from historical observations, and Online Tracking, which uses these parameters for real-time coordinate recognition.

Batch Initialization. Given the coordinates ($O^j_{c'^0}$ and $O^j_{c'^1}$) of spatial point O^j in the pixel coordinate systems c'^0 and c'^1, respectively, the relationship between $O^j_{c'^0}$ and $O^j_{c'^1}$ can be derived based on the principles of epipolar geometry[2], as follows:

$${O^j_{c'^1}}^T F_{c^1c^0} O^j_{c'^0} = 0. \tag{9}$$

To solve for the fundamental matrix $F_{c^1c^0}$, which describes the epipolar geometry between two views, a minimum of 7 pairs of corresponding points ($O^j_{c'^0}$ and $O^j_{c'^1}$) is required. However, 8 pairs are typically used to ensure a more stable and unique solution, especially when using the RANSAC algorithm to robustly estimate $F_{c^1c^0}$ in the presence of outliers. The fundamental matrix $F_{c^1c^0}$ is defined as:

$$F_{c^1c^0} = K^{1^{-T}} E_{c^1c^0} K^{0^{-1}}, \tag{10}$$

where K^0 and K^1 are the intrinsic parameter matrices of camera 0 and 1, respectively. The essential matrix $E_{c^1c^0}$, which relates corresponding points between two calibrated views and encodes the relative rotation and translation from c^1 to c^0, is given by:

$$E_{c^1c^0} = [t_{c^1c^0}]_\times R_{c^1c^0}. \tag{11}$$

In this equation, $[t_{c^1c^0}]_\times$ is the skew-symmetric matrix of the translation vector $t_{c^1c^0}$. The essential matrix $E_{c^1c^0}$ can be decomposed using singular value decomposition (SVD) and we define the extracted $t_{c^1c^0}$ as $\tilde{t}_{c^1c^0}$ and the extracted $R_{c^1c^0}$ as $\tilde{R}_{c^1c^0}$.

Given that multiplying $F_{c^1c^0}$ by any constant still satisfies Equation 9, there is a scale ambiguity in both $F_{c^1c^0}$ and $E_{c^1c^0}$. For a rotation matrix R, given its inherent property $\det(R) = 1$, we have $\tilde{R}_{c^1c^0} = R_{c^1c^0}$. For $\tilde{t}_{c^1c^0}$, there exists a scale factor s such that

$$\tilde{t}_{c^1c^0} = s t_{c^1c^0}. \tag{12}$$

From Equation 3 in the main text, the coordinates of a point P from c^0 to c^1 can be transformed by

$$P_{c^1} = R_{c^1c^0}P_{c^0} + t_{c^1c^0}. \tag{13}$$

Substituting $R_{c^1c^0}$ and $t_{c^1c^0}$ into Equation 13, we have:

$$sP_{c^1} = s\tilde{R}_{c^1c^0}P_{c^0} + \tilde{t}_{c^1c^0}. \tag{14}$$

Now, we define a scaled coordinate system $\hat{c}^i$ for each camera coordinate system c^i with a scale factor s. The coordinates of a point P from c^i to $\hat{c}^i$ are transformed by:

$$P_{\hat{c}^i} = sP_{c^i}. \tag{15}$$

Thus, Equation 14 can be rewritten as:

$$P_{\hat{c}^1} = \tilde{R}_{c^1c^0}P_{\hat{c}^0} + \tilde{t}_{c^1c^0}. \tag{16}$$

Therefore, based on the definition of similarity transformation between coordinates in different coordinate systems,

$$R_{\hat{c}^1\hat{c}^0} = \tilde{R}_{c^1c^0} = R_{c^1c^0}, \tag{17}$$

$$t_{\hat{c}^1\hat{c}^0} = \tilde{t}_{c^1c^0} = st_{c^1c^0}. \tag{18}$$

From Equation 7 in the main text, we can derive the expression for $O^j_{c^0}$:

$$O^j_{c^0} = -\frac{[O_{c'^1}]_\times K^1 R_{c^1c^0} {K^0}^{-1} O_{c'^0} \cdot [O_{c'^1}]_\times K^1 t_{c^1c^0}}{[O_{c'^1}]_\times K^1 R_{c^1c^0} {K^0}^{-1} O_{c'^0} \cdot [O_{c'^1}]_\times K^1 R_{c^1c^0} {K^0}^{-1} O_{c'^0}} {K^0}^{-1} O^j_{c'^0}. \tag{19}$$

Substituting Equation 15, Equation 17 and Equation 18 into Equation 19, we obtain

$$O^j_{\hat{c}^0} = -\frac{[O_{c'^1}]_\times K^1 \tilde{R}_{c^1c^0} {K^0}^{-1} O_{c'^0} \cdot [O_{c'^1}]_\times K^1 \tilde{t}_{c^1c^0}}{[O_{c'^1}]_\times K^1 \tilde{R}_{c^1c^0} {K^0}^{-1} O_{c'^0} \cdot [O_{c'^1}]_\times K^1 \tilde{R}_{c^1c^0} {K^0}^{-1} O_{c'^0}} {K^0}^{-1} O^j_{c'^0}. \tag{20}$$

Given the coordinates $O^j_{\hat{c}^0}$ of the spatial observed points O^j in $\hat{c}^0$ and coordinates $O^j_{c'^i}$ in the pixel coordinate systems c'^i of cameras $i = 2, 3, ..., N$, the rotation matrix $R_{\hat{c}^i\hat{c}^0}$ and translation vector $t_{\hat{c}^i\hat{c}^0}$ can be determined based on the Efficient Perspective-n-Point algorithm[3]. The EPnP method represents these points as a linear combination of four virtual control points and uses SVD to solve these linear equations.

Next, we calculate $R_{w\hat{c}^0}$ and $t_{w\hat{c}^0}$. From Equation 4 in the main text and Equation 15, we can derive the coordinate transformation of a spatial point P from w to $\hat{c}^0$:

$$P_{\hat{c}^0} = sR_{c^0w}P_w + t_{\hat{c}^0w}. \tag{21}$$

For the cameras, Equation 21 can be rewritten:

$$G^i_{\hat{c}^0} = sR_{c^0 w} G^i_w + t_{\hat{c}^0 w}. \quad (22)$$

Here, G^i_w is known. $G^i_{\hat{c}^0}$ represents the coordinates of camera i in the coordinate system $\hat{c}^0$, and $t_{\hat{c}^0 \hat{c}^i}$ as well. Based on the inverse operation of Equation 3 in the main text, $t_{\hat{c}^0 \hat{c}^i}$ can be calculated by:

$$G^i_{\hat{c}^0} = t_{\hat{c}^0 \hat{c}^i} = -{R_{\hat{c}^i \hat{c}^0}}^{-1} t_{\hat{c}^i \hat{c}^0}. \quad (23)$$

We use the Kabsch algorithm[4] for solving the similarity transformation to determine s, $R_{\hat{c}^0 w}$, and $t_{\hat{c}^0 w}$ in Equation 22. First we calculate the centroid $\overset{\circ}{G}_w$ and $\overset{\circ}{G}_{\hat{c}_0}$ for G^i_w and $G^i_{\hat{c}_0}$:

$$\overset{\circ}{G}_w = \frac{1}{N} \sum_{i=0}^{N-1} G^i_w,$$

$$\overset{\circ}{G}_{\hat{c}_0} = \frac{1}{N} \sum_{i=0}^{N-1} G^i_{\hat{c}_0}.$$

Let

$$\overset{*}{G^i_w} = G^i_w - \overset{\circ}{G}_w,$$

$$\overset{*}{G^i_{\hat{c}_0}} = G^i_{\hat{c}_0} - \overset{\circ}{G}_{\hat{c}_0},$$

$$H = \sum_{i=0}^{N-1} \overset{*}{G^i_{\hat{c}_0}} (\overset{*}{G^i_w})^T.$$

Find the SVD of H

$$H = U \Lambda V^T,$$

Then, $R_{\hat{c}^0 w}$, s and $t_{\hat{c}^0 w}$ can be computed as follows:

$$R_{\hat{c}^0 w} = V U^T,$$

$$s = \sqrt{\frac{\sum_{i=0}^{N} \|\overset{*}{G^i_{\hat{c}_0}}\|^2}{\sum_{i=0}^{N} \|\overset{*}{G^i_w}\|^2}},$$

$$t_{\hat{c}^0 w} = G^i_{\hat{c}^0} - sR_{c^0 w} G^i_w.$$

Online Tracking. Once the camera poses are determined during initialization, real-time coordinate recognition reduces to a direct coordinate transformation. Given the parameters s, $R_{\hat{c}^0 w}$, and $t_{\hat{c}^0 w}$ from Batch Initialization, the world coordinate O^j_w of the observed object O^j at time step j is obtained by applying the inverse of Equation 21:

$$O_{\mathrm{w}}^{j} = \frac{R_{\hat{\mathrm{c}}^0\mathrm{w}}{}^{-1}(O_{\hat{\mathrm{c}}^0}^{j} - t_{\hat{\mathrm{c}}^0\mathrm{w}})}{s}. \tag{24}$$

S3.3 Detailed Metrics

Metrics for 2D object detection model. To comprehensively evaluate the performance of the object detection model, we introduce a set of metrics that consider different IoU thresholds for Precision (P), Recall (R), and F1-score (F1). Before defining these metrics, we first clarify the basic concepts of True Positives (TP), False Positives (FP), True Negatives (TN), and False Negatives (FN):

True Positives ($\mathrm{TP}_{\mathrm{IoU}}$): The number of instances where the model correctly predicts an object and the predicted bounding box has an IoU with the ground truth bounding box that exceeds a specified IoU threshold.

False Positives ($\mathrm{FP}_{\mathrm{IoU}}$): The number of instances where the model incorrectly predicts an object where there is none, or the predicted bounding box does not meet the IoU threshold with any ground truth bounding box.

True Negatives ($\mathrm{TN}_{\mathrm{IoU}}$): The number of frames where the model correctly identifies the absence of objects.

False Negatives ($\mathrm{FN}_{\mathrm{IoU}}$): The number of instances where the model fails to detect objects that are actually present.

It should be noted that in cases where there is a UAV in the frame, but the detected bounding box has an IoU with the ground truth bounding box that is less than the specified IoU threshold, this instance is considered both a False Positive (FP) and a False Negative (FN).

With these definitions in place, we can now define the evaluation metrics:

IoU-Weighted Precision (IoU-P): Average Precision across IoU thresholds at every 5% increment from 50% to 95%:

$$\text{IoU-P} = \frac{1}{10}\sum_{k=0}^{9} P_{50+5k}, \tag{25}$$

where P_{IoU} is the proportion of correctly identified UAVs among all detections:

$$P_{\mathrm{IoU}} = \frac{\mathrm{TP}_{\mathrm{IoU}}}{\mathrm{TP}_{\mathrm{IoU}} + \mathrm{FP}_{\mathrm{IoU}}}. \tag{26}$$

IoU-Weighted Recall (IoU-R): Average Recall across IoU thresholds at every 5% increment from 50% to 95%:

$$\text{IoU-R} = \frac{1}{10}\sum_{k=0}^{9} R_{50+5k}, \tag{27}$$

where R_{IoU} is the proportion of correctly identified UAVs among all actual UAV samples:

$$R_{\mathrm{IoU}} = \frac{\mathrm{TP}_{\mathrm{IoU}}}{\mathrm{TP}_{\mathrm{IoU}} + \mathrm{FN}_{\mathrm{IoU}}}. \tag{28}$$

IoU-Weighted F1-Score (IoU-F1): Average F1-score across IoU thresholds at every 5% increment from 50% to 95%:

$$\text{IoU-F1} = \frac{1}{10}\sum_{k=0}^{9} F1_{50+5k}, \tag{29}$$

where $F1_{\text{IoU}}$ is the harmonic mean of Precision and Recall:

$$F1_{\text{IoU}} = 2 \cdot \frac{P_{\text{IoU}} \cdot R_{\text{IoU}}}{P_{\text{IoU}} + R_{\text{IoU}}}. \tag{30}$$

Metrics for 3D coordinate recognition. To evaluate the accuracy of coordinate recognition for the flying object, we employ several metrics:

Root Mean Square Error (RMSE):

$$\text{RMSE} = \sqrt{\frac{\sum_{j=0}^{M-1} \left\| \tilde{O}_{\text{w}}^{j} - O_{\text{w}}^{j} \right\|_2^2}{M}}. \tag{31}$$

Mean Absolute Error (MAE):

$$\text{MAE} = \frac{1}{M}\sum_{j=0}^{M-1} \left\| \tilde{O}_{\text{w}}^{j} - O_{\text{w}}^{j} \right\|_1. \tag{32}$$

Maximum Error:

$$\text{Max Error} = \max_{j=0}^{M-1} \left\| \tilde{O}_{\text{w}}^{j} - O_{\text{w}}^{j} \right\|_2. \tag{33}$$

R-squared (Coefficient of Determination):

$$R^2 = 1 - \frac{\sum_{j=0}^{M-1} \left\| \tilde{O}_{\text{w}}^{j} - O_{\text{w}}^{j} \right\|_2^2}{\sum_{j=0}^{M-1} \left\| O_{\text{w}}^{j} - \bar{O}_{\text{w}} \right\|_2^2}. \tag{34}$$

Here, $\tilde{O}_{\text{w}}^{j}$ and O_{w}^{j} represent the computed and ground truth spatial point coordinates in the world coordinate system at time step j, respectively, and M denotes the total number of time steps.

S3.4 Preparation for UAV Coordinate Recognition

After obtaining the detection boxes for all cameras at each time step, we calculate the center coordinates of these detection boxes as the UAV 2D coordinates in the pixel coordinate systems of the three cameras.

Preprocessing Requirements. The 2D pixel coordinates of the UAV captured by each camera at each time step cannot be directly used for 3D coordinate recognition due to several factors: geometric distortion from the lens, undetermined 3D coordinates of the cameras in the world coordinate system, unknown intrinsic camera parameters,

and lack of time synchronization between the image series from different cameras. To address these issues, preliminary steps include calibrating the intrinsic parameters and correcting for distortion using Zhang's method[5], determining the camera coordinates in the world coordinate system with a GNSS receiver, and synchronizing the image time series from different cameras.

Calibration and Synchronization. Using the distortion parameters of the camera, we correct the deviations in the 2D pixel coordinates of the UAV. Following this, the time correspondence between different cameras is established, allowing us to convert the 2D pixel coordinate time series from the original camera-based time to a unified global time.

Multi-camera Time Alignment. During Phase I (Batch Initialization), the 2D coordinate time series of the UAV obtained from camera 0 and camera 1 are unaligned due to the discrete nature of image capture. To align these time series, we smooth the UAV 2D coordinate time series from camera 1 using cubic spline interpolation[6] and resample it at the time points of time series from camera 0, generating an aligned timeline. For the remaining cameras i (where $N > i \geq 2$; $i = 2$ and $N = 3$ in the UAV experiment), we identify the overlapping time periods between the initial 3D trajectory and the time when camera i observes the UAV. We then interpolate the 2D coordinate time series from camera i to match the time points of the initial 3D trajectory. During Phase II (Online Tracking), frames are matched directly by timestamp without interpolation.

S4 Supplementary Note 1

In the numerical simulations, the camera positioning error levels are set to $0.1, 0.2, 0.4, 0.6, 0.8, 1.0\,\mathrm{m}$. In engineering scenarios, cameras use high-precision Real-Time Kinematic (RTK) GNSS devices for positioning, which involve two main sources of error: a 10 cm installation error between the GNSS receiver and the camera, and the GNSS receiver positioning error, which is less than 10 cm[7].

The pixel deviation levels in the numerical simulations are set to $1, 2, 4, 6, 8, 10$ pixels. Consider a standard camera with a resolution of 1920×1080 pixels, no geometric distortion, and an optical axis centered in the image, with focal lengths of 1000 in both the x and y directions. For a sphere with a diameter of 0.5 m located along the camera principal axis, the sphere occupies 20 pixels when it is 25 m away, 10 pixels at 50 m, and 2 pixels at 250 m in both the x and y directions. The 2D coordinates of this target in the image are defined by the center of the occupied pixels. The deviation between any pixel on the occupied area and the center pixel position is half the length of the occupied pixels and is varied between 1 and 10 pixels.

The scale factors for the scene in the numerical simulations are set to $0.25, 0.5, 1, 1.5$, and 2, scaling the original scene size of $200 \times 200 \times 100\,\mathrm{m}$ to a range from $50 \times 50 \times 25\,\mathrm{m}$ to $400 \times 400 \times 200\,\mathrm{m}$. Here, we set a fixed camera positioning error of 0.2 m and a pixel deviation of 3 pixels. For static camera positioning, we consider the camera positioning error is 0.2 m, which includes a 10 cm positioning error from the high-precision RTK GNSS receiver and an additional 10 cm installation error. For pixel deviations, we consider a 0.5 m spherical object. When it is positioned along the

camera's principal axis at a distance of 83.3 m from the optical center, it occupies 6 pixels in both the x and y directions, with a maximum pixel deviation of 3 pixels. While the number of pixels occupied by the object would vary at different distances, we simplify the experimental parameters by setting the pixel deviations to a fixed value of 3 pixels.

S5 Supplementary Note 2

The camera parameters, including resolution, frame rate, intrinsic parameters, distortion coefficients, position, corresponding frames, and unified time, depend on the specific camera model (as detailed in Supplementary Table 3).

The resolution and frame rate are obtained from the technical specifications of the Huawei camera devices used in the experiment, with 1080p resolution (1920×1080 pixels) and a frame rate of 30 fps.

The intrinsic parameters and distortion coefficients are estimated using Zhang's method[5], a widely recognized approach for camera calibration. This method involves capturing a series of images from different angles to accurately calculate these parameters. The calibration tool used is a calibration plate, as shown in Supplementary Figure 3, where each square on the plate measures 30.5 mm. During calibration, the plate is positioned at various orientations and distances from the camera, ensuring a comprehensive calibration across different perspectives.

Camera positions are measured using a high-precision RTK GNSS receiver, providing each camera position within the world coordinate system with an accuracy within 10 cm. We use WGS84 as our geodetic reference ellipsoid, which provides components along the X, Y, and Z axes in the Cartesian coordinate system. However, an installation error also exists between the GNSS receiver and the camera optical center, estimated at approximately 10 cm. These high-precision RTK GNSS devices are connected to a local Continuously Operating Reference Stations (CORS) network, which provides GNSS positioning services across the area. The CORS network enables RTK GNSS systems to achieve 3D positioning accuracy of approximately ± 3 cm under optimal conditions[7].

We establish the relationship between video frames and the time reference (Beijing time in this case) for camera time synchronization by having each camera continue recording while capturing the Beijing time. Camera 0 records frame 20368 at 07 : 09 : 42 Beijing time. Similarly, camera 1 records frame 18712 at 07 : 08 : 40, and camera 2 records frame 17370 at 07 : 07 : 54.

S6 Supplementary Note 3

YOLOv12 (You Only Look Once version 12) is a state-of-the-art anchor-free object detection model released in February 2025, jointly proposed by the University at Buffalo and the University of Chinese Academy of Sciences. As a newly developed member of the YOLO family, YOLOv12 is designed to achieve both high detection accuracy and lightweight efficiency, making it particularly well-suited for real-time applications such as UAV monitoring in time-series imagery. Distinct from previous YOLO variants, YOLOv12 introduces several architectural innovations, including the

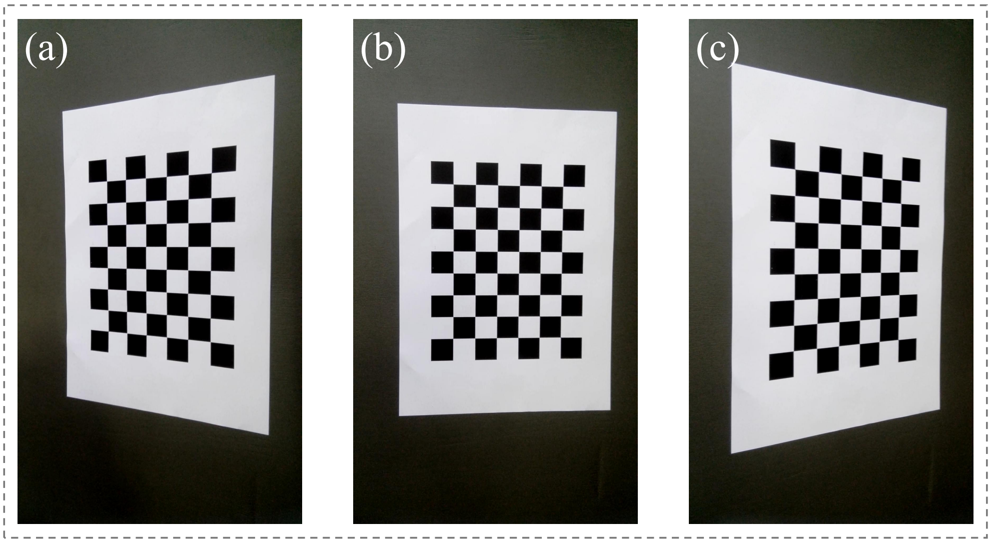

Supplementary Figure 3 Calibration plate used for intrinsic parameter calibration. The plate features a 7×13 grid with each square measuring 30.5 mm. (a), (b), and (c) show the calibration plate captured from different viewing angles. Photos taken by the author Junfan Yi.

Supplementary Table 3 Parameters for multiple camera models. All three cameras are Huawei devices with 1920×1080 resolution at 30 FPS.

Camera Index	Intrinsic Parameters Matrix	Distortion Coefficients	Position (m)	Corresponding Frames	Unified Time
0	$\begin{bmatrix} 1557.6 & 0 & 952.8 \\ 0 & 1557.3 & 555.5 \\ 0 & 0 & 1 \end{bmatrix}$	$\begin{bmatrix} 0.0418 \\ 0.3660 \\ 0.0027 \\ -0.0001 \\ -1.9523 \end{bmatrix}$	139.64<br>157.54<br>98.15	20368	07:09:42
1	$\begin{bmatrix} 1563.7 & 0 & 943.9 \\ 0 & 1564.1 & 557.8 \\ 0 & 0 & 1 \end{bmatrix}$	$\begin{bmatrix} 0.0084 \\ 0.9725 \\ 0.0039 \\ -0.0028 \\ -4.3086 \end{bmatrix}$	156.03<br>101.51<br>188.90	18712	07:08:40
2	$\begin{bmatrix} 1574.3 & 0 & 958.5 \\ 0 & 1574.5 & 544.4 \\ 0 & 0 & 1 \end{bmatrix}$	$\begin{bmatrix} 0.0130 \\ 0.9604 \\ 0.0024 \\ -0.0023 \\ -4.1495 \end{bmatrix}$	210.67<br>138.01<br>180.16	17370	07:07:54

A2C2f module for enhanced feature representation and an efficient decoupled head design. These improvements streamline the detection pipeline, reduce computational overhead through depthwise separable convolutions, and enhance end-to-end feature learning for more accurate object localization and classification. These enhancements result in faster convergence and improved inference performance while maintaining a compact model size. The network structure is divided into three parts: the backbone, the neck, and the head, as shown in Supplementary Figure 4.

Characteristics of YOLOv12.

- R-ELAN Backbone with Layer Scaling: YOLOv12 employs an R-ELAN structure with deep residual links and bottleneck expansion. This design improves training stability and depth efficiency, enabling stronger feature extraction without substantially increasing computational cost.
- FlashAttention for Efficient Attention: By integrating area-based FlashAttention, YOLOv12 reduces memory access overhead and accelerates inference. The attention modules enhance focus on salient regions, improving performance in cluttered or complex scenes.
- Optimized Neck Design for Multi-scale Fusion: The neck uses depthwise separable layers and enhanced aggregation strategies to better fuse features across scales. This leads to improved detection of objects of various sizes with lower inference latency.
- Lightweight and Deployment-Friendly Architecture: YOLOv12 streamlines the network with pruning, quantization, and efficient convolution blocks. It delivers competitive accuracy with fewer parameters, making it suitable for real-time and edge applications such as UAV detection.

Training detail The hardware setup includes dual Intel(R) Xeon(R) Gold 6430 CPUs (128 threads total) and an NVIDIA GeForce RTX 4090 with 24GB of memory. The software environment consists of PyTorch v2.3.0 running on a Linux system with CUDA 12.1. For end-to-end network optimization, we use the SGD algorithm with the official YOLOv12n pretrained weights, and the following hyperparameters:

The learning rate is set to 0.01, weight decay to 0.0005, momentum to 0.937, and the batch size to 48. The input image resolution is fixed at 960×960 pixels. We apply a scale factor of 0.5, enable full mosaic augmentation, and use moderate copy-paste augmentation (0.1), while disabling mixup. Training runs for 300 epochs, with early stopping triggered if performance does not improve after 30 epochs. All other configurations remain as the default settings of the original YOLOv12 model.

S7 Supplementary Note 4

S7.1 YOLOv12-TS Parameters

Detection. The YOLOv12n model uses an input resolution of $960 \times 960 \times 3$. During inference, a confidence threshold of 0.25 and an IoU threshold of 0.7 are applied. The model is trained with a batch size of 48 for 300 epochs.

Outlier Rejection. For temporal consistency, a detection pair at frames i and j is valid if the displacement $d(i,j) < 10 \times (j-i)$ pixels, with a maximum frame gap

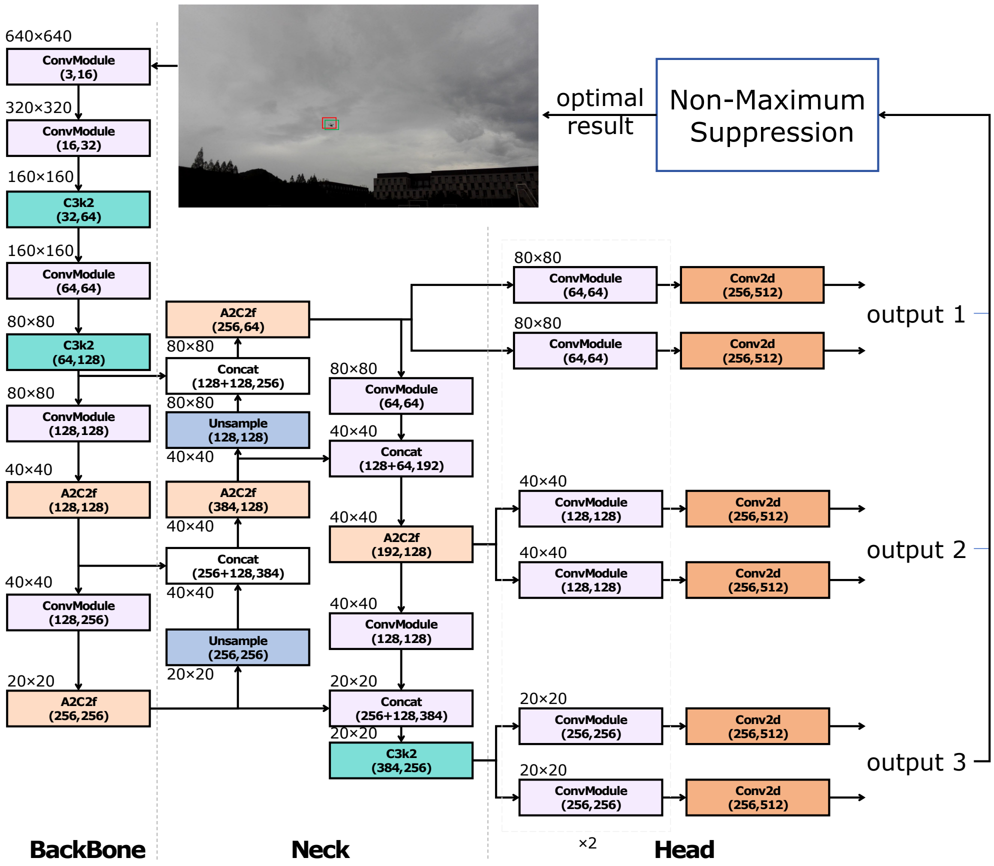

Supplementary Figure 4 Overview of the network architecture of YOLOv12. (a) Backbone: The backbone is the core component responsible for extracting multi-scale feature representations from the input image. YOLOv12 adopts a lightweight yet expressive backbone composed of Conv modules, C3k2 blocks, and the newly proposed A2C2f modules. The C3k2 module is a refined version of the C3 block, integrating three convolutional layers and two residual branches. This design enhances feature reuse and enriches intermediate representations while maintaining a compact structure. The A2C2f module incorporates region-aware attention mechanisms within a convolutional framework. It divides the feature map into multiple regions to capture long-range dependencies efficiently and maintains a large receptive field with minimal computational overhead. (b) Neck: The neck of YOLOv12 adopts a lightweight feature fusion strategy composed of Upsample, Concat, and the A2C2f modules. This design facilitates the integration of multi-scale features extracted from the backbone, allowing the model to better capture spatial and semantic information across different resolutions. The A2C2f modules further refine the fused features using region-aware attention, enhancing detection performance while maintaining efficiency. (c) Head: The head of YOLOv12 adopts a decoupled structure, consistent with YOLO11, in which classification and localization branches are designed and optimized independently. Photo taken by the author Junfan Yi.

of 3. During Phase II, the outlier rejection looks back at most 10 frames, rejecting detections exceeding 10 times the expected displacement.

Trajectory Completion. Linear interpolation fills gaps of up to 20 frames between confirmed detections. An edge margin of 1% from the image border distinguishes center and edge regions. The speed threshold is 2.5 times the IQR-filtered maximum speed from confirmed detections.

S7.2 Dual-Phase Strategy Parameters

Batch Initialization. The system buffers $N = 2000$ frames before triggering pose estimation. Detections within 50 pixels of the image boundary are excluded.

Online Tracking. A larger edge margin of 100 pixels is applied. Frames are rejected if the single-camera reprojection error exceeds 25 pixels or the mean reprojection error exceeds 20 pixels.

Bundle Adjustment Backend. The background refinement module runs every 2000 frames after initialization. Optimization uses the Levenberg-Marquardt algorithm with Huber loss ($\delta = 4.0$). Camera 0 is fixed as the reference frame.

S7.3 Geometric Solver Parameters

The fundamental matrix is estimated using RANSAC with a reprojection threshold of 15 pixels. Camera poses for additional views are computed using the EPnP algorithm with RANSAC, with a reprojection error threshold of 8 pixels.

S8 Supplementary Note 5

To validate the theoretical precision of our SVD-based framework under idealized conditions, we conduct a batch-mode simulation which is not real-time. Unlike the dual-phase strategy in the main text, all 300 frames are processed jointly without phase separation, representing the theoretical upper bound of accuracy (Supplementary Figure 5).

The results show that errors are almost negligible, where the RMSE is 3.2×10^{-4} m, the MAE is 3.2×10^{-4} m, the Maximum Error is 4.9×10^{-4} m, and the R-squared is almost 1.

To evaluate the impact of engineering factors on 3D coordinate recognition accuracy, we perform simulations with camera positioning errors, pixel detection deviations, and scene scaling (Supplementary Figure 6; Supplementary Note 1).

For 3D coordinate recognition with camera positioning deviations (Supplementary Figure 6(a)) of 0.1 m, 0.2 m, 0.4 m, 0.6 m, 0.8 m, and 1.0 m, the RMSE are 0.0777 m, 0.1553 m, 0.3109 m, 0.4642 m, 0.6162 m, and 0.7673 m, respectively, while the MAE are 0.0763 m, 0.1525 m, 0.3053 m, 0.4558 m, 0.6050 m, and 0.7533 m, respectively. The Maximum Error for these deviations are 0.1004 m, 0.2004 m, 0.4012 m, 0.6004 m, 0.7965 m, and 0.9936 m, respectively. As the camera positioning error increases, the error in spatial point coordinate recognition also increases.

The RMSE for pixel deviations (Supplementary Figure 6(b)) of 1, 2, 4, 6, 8, and 10 pixels are 1.3811 m, 1.3465 m, 1.8805 m, 2.8155 m, 3.4120 m, and 3.7553 m, respectively,

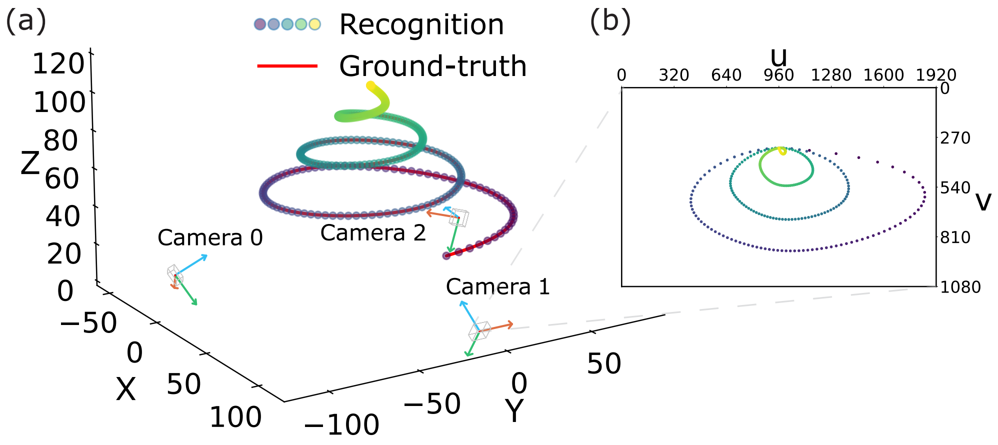

Supplementary Figure 5 The 3D scene of the batch-mode numerical simulation. (a) The red ascending spiral trajectory over 300 time steps, with gradient-colored dots representing the calculated spatial points. Three ground cameras, each located 100 m from the center, are positioned at 120° intervals. (b) The projection of the spatial point onto the image plane of camera 1.

while the MAE are 1.3545 m, 1.3115 m, 1.8195 m, 2.7303 m, 3.3034 m, and 3.6272 m, respectively. The Maximum Error for these pixel deviations are 2.0960 m, 2.1552 m, 3.1625 m, 4.6152 m, 5.6120 m, and 6.2098 m, respectively. It is evident that pixel deviations have a much greater impact on spatial point coordinate recognition compared to camera positioning errors.

Supplementary Figure 6(c) depicts the errors in the calculated 3D coordinates for each scaling factor, with RMSE of 0.3942 m, 0.7316 m, 1.4404 m, 2.1382 m, and 2.9114 m, and corresponding MAE of 0.3829 m, 0.7088 m, 1.3940 m, 2.0694 m, and 2.8185 m for scaling factors of 0.25, 0.5, 1, 1.5, and 2, respectively. The Maximum Error for these scaling factors are 0.6415 m, 1.2189 m, 2.4168 m, 3.5901 m, and 4.8698 m, respectively. When both camera positioning errors and pixel deviations are considered, the larger the spatial scene, the greater the coordinate recognition errors.

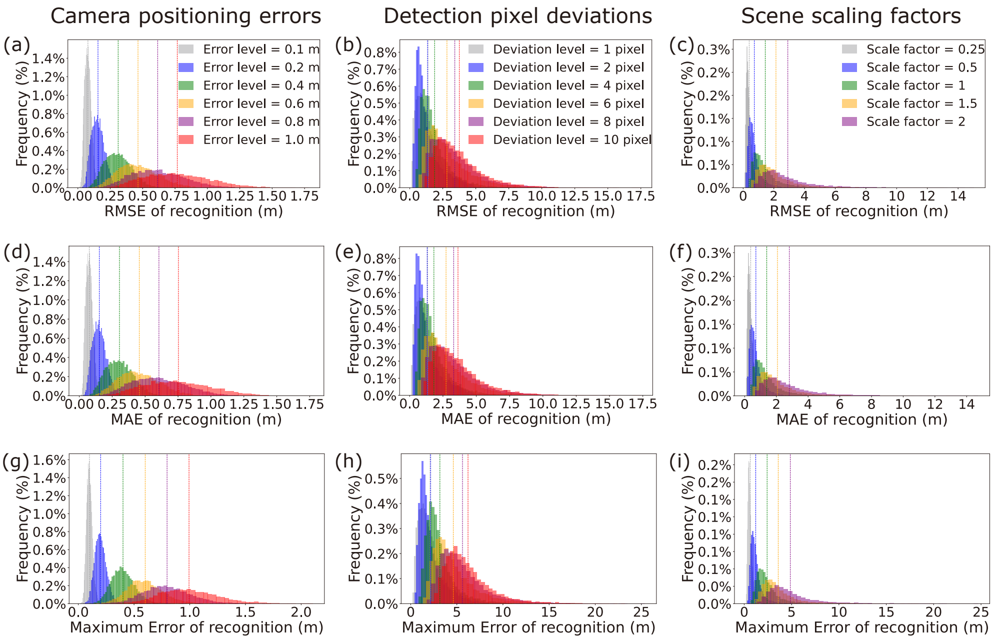

Supplementary Figure 6 Error histograms of coordinate recognition under different perturbations. For each perturbation level, 10,000 experiments are conducted. (a), (d), (g) Camera positioning errors: Histograms show the distribution of coordinate recognition errors across 10,000 simulations for each positioning error level ($e_p = 0.1, 0.2, 0.4, 0.6, 0.8, 1.0$ m) for RMSE (a), MAE (d), and Maximum Error (g). (b), (e), (h) Pixel deviations: Histograms display the distribution of coordinate recognition errors across 10,000 simulations for each pixel deviation level ($e_{\text{pix}} = 1, 2, 4, 6, 8, 10$ pixels) for RMSE (b), MAE (e), and Maximum Error (h). (c), (f), (i) Scene scaling factors: The scene is scaled using factors of 0.25, 0.5, 1, 1.5, and 2, with a camera positioning error of 0.2 m and a random pixel deviation of 3 pixels, for RMSE (c), MAE (f), and Maximum Error (i). A scaling factor of 0.25 implies that the coordinates of both the spatial points and the camera positions are scaled to 25% of their original values.

References

[1] Sie, N. J., Srigrarom, S. & Huang, S. *Field test validations of vision-based multi-camera multi-drone tracking and 3D localizing with concurrent camera pose estimation.* In *Proc. 2021 IEEE 6th International Conference on Control and Robotics Engineering (ICCRE)*, 139–144 (IEEE, 2021).

[2] Longuet-Higgins, H. C. A computer algorithm for reconstructing a scene from two projections. *Nature* **293**, 133–135 (1981).

[3] Lepetit, V., Moreno-Noguer, F. & Fua, P. EPnP: An accurate $O(n)$ solution to the PnP problem. *International Journal of Computer Vision* **81**, 155–166 (2009).

[4] Kabsch, W. A discussion of the solution for the best rotation to relate two sets of vectors. *Acta Crystallographica Section A: Crystal Physics, Diffraction, Theoretical and General Crystallography* **34**, 827–828 (1978).

[5] Zhang, Z. A flexible new technique for camera calibration. *IEEE Transactions on Pattern Analysis and Machine Intelligence* **22**, 1330–1334 (2000).

[6] Schoenberg, I. J. Contributions to the problem of approximation of equidistant data by analytic functions: Part A. On the problem of smoothing or graduation. A first class of analytic approximation formulae. *Quarterly of applied mathematics* **4**, 45–99 (1946).

[7] Alkan, R. M., Erol, S., İlçi, V. & Ozulu, İ. M. Comparative analysis of real-time kinematic and PPP techniques in dynamic environment. *Measurement* **163**, 107995 (2020).
